# Evaluation of the effect of the internal layer of oak fruit (jaft) extract on the prevention of gastric ulcers caused by stress in male rats

**DOI:** 10.25122/jml-2017-0025

**Published:** 2018

**Authors:** Mehrzad Jafari Barmak, Alireza Dehghan menshadi, Reza Mahmoudi, Hassan Bardania

**Affiliations:** 1. Cellular and Molecular Research Center, Yasuj University of Medical Sciences, Yasuj, Iran

**Keywords:** Jaft, Stomach ulcer, Stress, Nitric oxide, Malondialdehyde

## Abstract

**Introduction:** Many drugs with high efficacy are used to treat stomach ulcers, but they have many side effects. Therefore, much effort has been made to find new effective compounds from plant extracts. The aqueous extract of the internal layer of oak fruit (jaft) contains antioxidants and tannins; it has many desirable properties such as inhibition of the growth of pathogens. In this study, the preventing effect of Jaft extract on stomach ulcers induced by stress was investigated.

**Matherials and Methods:** The effect of the extract on the prevention of gastric ulcer was investigated using a variety of methods including pH measurement, nitric oxide (NO), malondialdehyde (MDA), and histological methods. Rats were randomly divided into six groups. Five groups were gavage fed with different concentrations of Jaft extract, ranitidine and normal saline. The sixth group was gavage fed with normal saline as the control group. Ranitidine and normal saline were used as positive and negative controls, respectively. Data were analyzed by one-way analysis of variance (ANOVA) followed by Tukey’s post hoc test using SPSS version 18.0.

**Results:** It was revealed that the average thickness of the mucous glands and mucosal folds in the groups receiving the extract (250, 500 and 750 mg/kg) did not significantly decrease when compared with the situation of the control group (p<0.05). However, the average nitric oxide (NO) and malondialdehyde (MDA) in the control group meaningfully decreased in comparison with groups receiving the extract (250, 500 and 750 mg/kg). The average pH in groups receiving the extract and ranitidine significantly increased compared to the control group (p<0.05).

**Conclusions:** Jaft extract contains abundant polyphenolic compounds and tannins and has several biological properties such as pharmacological properties, antioxidant activity, and inhibition of lipid oxidation. Therefore, it has the potential to prevent and treat stomach ulcers.

## Introduction

Inflammation and ulcers are two conventional types of damage in the digestive tract. The primary cause of ulcers is the disruption of the integrity of the stomach mucous membrane, which is a barrier that protects its lower cells against damaging elements of domestic or foreign origins [1]. Various factors, such as increased secretion of acid and pepsin, decreased mucosal blood flow, reduced synthesis of prostaglandins, lower production of nitric oxide, and increased production of free radicals, stimulate the disruption of mucous membrane integrity [2]. The appearance of lesions or prevention of lesion formation depends upon the balance between aggressive agents (e.g., acid and pepsin secretions and leukotrienes), mucosal defensive factors, (e.g., blood flow, prostaglandins) and factors producing energy. Various internal or external harmful factors, including high alcohol consumption, smoking, excessive stress, and long-term use of non-steroidal anti-inflammatory drugs, can destroy this balance and cause mucosal ulceration in the stomach [3, 4].

Excessive stress and free radicals, such as reactive oxygen species (ROS), produced by many natural metabolic processes are one of the most important factors to induce stomach ulcer. Although various enzymes, such as superoxide dismutase, catalase, and glutathione peroxidase, remove the radicals, any deficiency in the enzymes’ functions can increase the amount of ROS in the body. The antioxidant molecules may have a protective role in preventing gastric mucosal injury by removing the free radicals and preventing lipid peroxidation [5, 6]. Although there are many chemical drugs with high efficacy (e.g., omeprazole, metronidazole, and ranitidine) to treat stomach ulcer, these drugs are costly and have significant adverse side effects. Therefore, many studies have been conducted to find effective natural and herbal compounds to treat ulcer [5]. One of these compounds happens to be tannic acid, which can prevent the growth of pathogens [7] and accelerate wound healing and burn recovery [5, 8].

One of the herbal sources of tannic acid is the oak tree, a member of the Quercus genus and the Fagaceae family. The oak fruit contains significant amounts of active biological compounds including tannins, gallic acid, ellagic acid, among others, which have antioxidant properties [5, 8].

More studies have been conducted on the extracted compounds from fruits, leaves, roots, and skin, while others studies have been conducted in conjunction with the aquatic extracts of the internal layer of oak fruits (jaft). One of the mechanisms of the stress-induced lesions is the increase in the production of free radicals. This study was designed due to the presence of numerous antioxidant compounds in the jaft extract. Therefore, the effect of the jaft extract was used to prevent gastric ulcer induced by stress to investigate the effect of the extract on the prevention of gastric ulcer using a variety of methods including pH measurement, nitric oxide (NO), malondialdehyde (MDA), and histological methods.

## Experimental section

### Jaft preparation

500 gr of the internal layer of oak fruit was added to 2 L of distilled water and incubated at room temperature for 48 hours. Then, the mixture was filtered and the obtained extract was dried using an incubator at 40–45 °C. The median lethal dose (LD50) of Jaft extract was calculated at 5000 mg/kg by Mirzaee and colleagues at the Yasuj University of Medical Sciences [5, 7, 9].

### Animals

30 male Wistar rats (weighing approximately 180–200 g) were purchased from the Pasteur Institute (Tehran, Iran). The animals were acclimated to the animal facility of Yasuj University of Medical Sciences for 10 days, at a temperature of 24 ± 1 °C and humidity of 55 ± 5 %. The experimental procedures were approved by the Animal Care and Use Committee (Yasuj University of Medical Sciences).

### Protocol study

The rats were randomly divided into six groups of five animals each, and after the period of adaptation, the five groups starved for 48 hours. In order to avoid dehydration during the period of starving, they were fed with 8% sucrose in 2% salt solution. The five groups, which were deprived of food for 48 hours, were gavage fed with different concentrations of Jaft extract (the first, second and third groups with 250, 500, 750 mg/kg of extract, respectively), ranitidine (the fourth group with 150 mg/kg) and normal saline (the fifth group as control). The sixth group was gavage fed with normal saline as the control group.

Two hours after receiving the extracts, the induction of gastric mucosal lesions was performed in the rats in groups one to five via a method previously reported by Takagi et al. [10]. In this regard, the animals were placed in a restraint device and immersed to the level of the xiphoid process in a water bath (20–22 °C) for 5 hours. The sixth group of rats, which did not need to stress, was kept in the cage. After induction, the rats were removed from the restraint device and weighed. Following that, all rats were anesthetized using ether and then dissected to evaluate the wounds. The gastroesophageal junction (cardia) was closed by forceps, then the stomach content was aspirated when 2 ml of distilled water was imported into the stomach and centrifuged. The supernatants were taken, and their pH was measured using a pH meter [11].

### Histological assessment

The affected gastric tissue was evaluated by a stereomicroscope to examine gastric ulcers. 0.5 mm² of damaged gastric tissue was transferred to a 10% buffered formalin solution for histological study and processed by a tissue-processing machine. A microtome (Leica) was then used to cut five micrometers from the paraffin blocks. The tissue slides were stained with hematoxylin-eosin stain and then evaluated using an optical microscope (Olympus BX51) [5]. Some parts of the stomach were taken and stored at -20°C to measure their NO and MDA levels.

### Measurement of nitrite production

The release of NO was examined using the method described by Garrat [12]. In this method, the nitrite and nitrate accumulation in the gastric tissue was assessed using a commercially available NO kit. The nitrate in the tissue is reduced to nitrite using the nitrate reductase enzyme. After reduction, the optical density value was measured at 540 nm using a UV spectrophotometer and compared to the standard curve for sodium nitrite.

### Malondialdehyde (MDA) measurement

The lipid peroxidation of gastric tissue was determined according to a previously described method [13]. As the end product of the lipid peroxidation, MDA chain reacts with thiobarbituric acid (TBA) to produce a colored product (TBA-MDA) which absorbs light at 535 nm in acidic solution. Briefly, the serum samples (500 μL) were mixed with 2 mL of the reaction solution containing 100 mL HCl 0.25 M, 15 gr trichloroacetic acid (TCA) and 375 mg TBA and then heated in boiling water for 15 minutes. After cooling, MDA concentrations were determined spectrophotometrically by the absorbance of TBA reactive substances at 532 nm.

### Statistical analysis

The results were presented as the mean ± standard deviation and the data were analyzed by one-way analysis of variance (ANOVA) followed by Tukey’s post hoc test using SPSS version 18.0 to determine whether the differences are statistically significant (*p < 0.05*).

## Results

**Figure 1:**
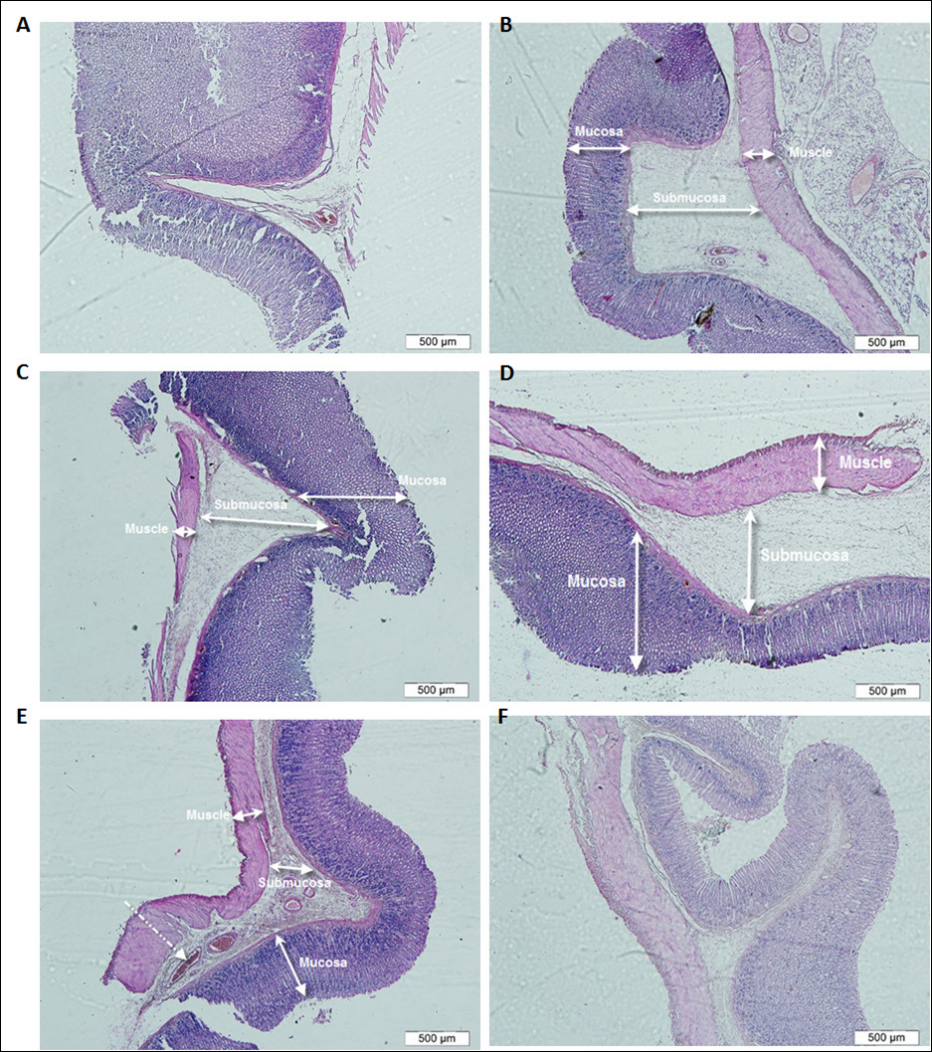
Cross sections of gastric tissues in healthy control (sixth group) (A), positive control group (fifth group) (B), group receiving 250 mg/kg Jaft extract (first group) (C), group receiving 500 mg/kg extract (second group) (D), group receiving 750 mg/kg extract (third group) (E) and group receiving ranitidine (Forth group) (F). (Coloring with Hematoxylin-Eosin, magnification 4x).

The effect of jaft extract on the prevention and treatment of stomach ulcer was investigated using various methods. There were no injuries visible to the naked eye in any of the groups. The swelling and inflammation in gastric tissues were only observed in some groups, including those receiving the extract (the first, second, and third groups) and the control group (fifth group). The present results reveal that the average thickness of the mucosa did not significantly decrease in the treated groups compared to the control group (P>0.05). The control group (fifth group) showed some signs of inflammation, including an increase in interstitial fluid volume (edema) and venous congestion in the submucosal layer, reduction in the length of the mucous glands, mucous folds, and bleeding in the mucous layer (Fig. 1B) compared to healthy gastric tissues (sixth group) without any submucosal edema or venous congestion (Fig. 1A).

Although venous congestion and submucosal edema were seen in the mucosal tissues of the groups receiving 250 and 500 mg/kg jaft extract (first, second groups) (Fig. 1C and D) respectively, the thickness of the mucosa and the structure of gastric tissues in the group receiving 750 mg/kg extract (third group) did not decrease compared to healthy tissues (Fig. 1E). In addition, more mucous glands and folds were observed in the gastric tissue of the group receiving ranitidine (fourth group) compared to healthy tissue (Fig. 1F). The results revealed that the jaft extract prevented stomach inflammation and ulcers.

**Figure 2:**
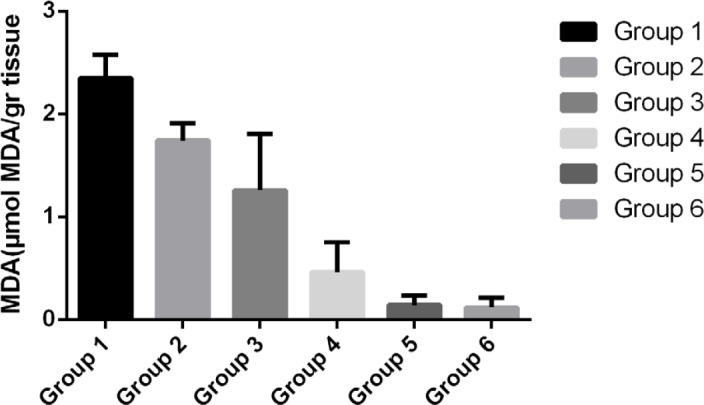
The average level of malondialdehyde (MDA) in the gastric tissue of various studied groups.

**Figure 3:**
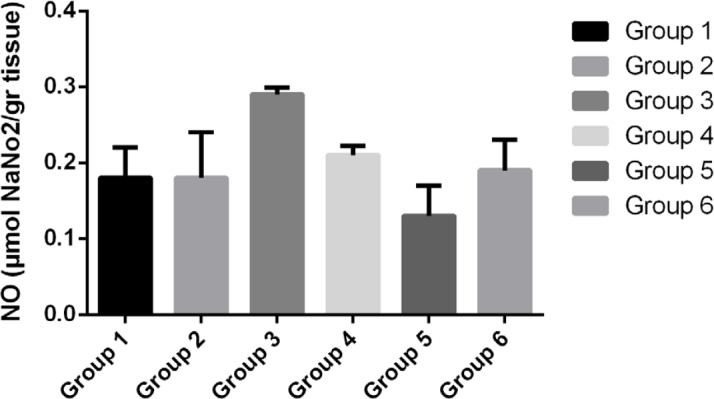
The average level of nitric oxide (NO) in the gastric tissue of various studied groups.

**Figure 4:**
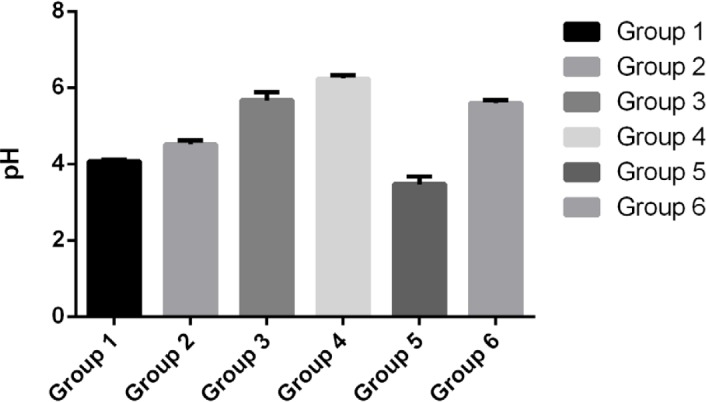
The average level of pH in the gastric tissue of various studied groups.

Table 1 demonstrates that the average thickness of mucous folds and mucous glands did not significantly increase in the treated groups when compared to the control group (P<0.05). However, the average level of MDA (Fig. 2), NO (Fig. 3), and pH (Fig. 4) meaningfully changed in the treated groups compared to the control group. The increase in the average level of MDA in groups receiving Jaft (first, second and third groups) is significant compared to the control group and the group receiving ranitidine. Although the results show that the level of MDA decreased with the increasing concentration of jaft extract, the MDA level in the third group with the highest jaft extract concentration (750 mg/kg) shows significant differences compared to the group receiving ranitidine.

The production of NO in the control group (fifth group) and the third group (receiving 750 mg/kg Jaft extract) significantly decreased and increased compared with other groups, respectively. The average pH in the third and fourth groups, which received 750mg/kg Jaft extract and ranitidine respectively, was maintained at approximately 5.6 and did not change compared to healthy mice (sixth group).

## Discussion

In the present study, the effect of jaft extract on gastrointestinal ulcer was investigated. The results show that the average thickness of the mucous glands in the group receiving ranitidine decreased compared to the group receiving the oak extract. The protective effect of the Bael fruit (*Aegle marmelos* [AM], family: *Rutaceae*) on aspirin-induced gastrointestinal lesions was previously evaluated by Shyamal K. Das *et al*. and they showed that the fruit improves wound areas and the mucosal thickness of the stomach. In addition, the use of ranitidine under stress significantly increased the surface and thickness of the wounds [13].

In this study, the thickness of the mucosal layer (edema) decreased in the groups receiving jaft extract (the first, second, and third groups). This can be attributed to the reduction of the fluids and attraction of proteins and water from the submucosal blood vessels.

Previous studies indicated that jaft extract has an antimicrobial effect and its therapeutic effect is related to the presence of tannic acid in the extract that attracts water and proteins from the intestine [14]. In addition, the thickness of the mucosal folds did not decrease in the groups receiving jaft extract (first, second, and third groups) compared to the control group (fifth group). It is an indicative of the stomach-protective effect of jaft extract.

**Table 1:** The average size of wound and thickness of the submucosa, mucous glands and mucous folds in the studied groups

Group	The wound surface (mm^2^)	Thickness of the mucous glands (μm)	Thickness of the mucous folds (μm)	Thickness of the submucosal area (μm)
First group	0	4.39 ± 1.97	8.19 ± 3.19	2.99 ± 1.88
Second group	0	3.91 ± 2.03	7.86 ± 3.22	3.32 ± 1.79
Third group	0	3.23 ± 1.37	6.78 ± 2.82	2.38 ± 1.55
Forth group	0	2.94 ± 1.71	7.86 ± 4.23	1.89 ± 1.25
Fifth group	0	2.77 ± 1.13	6.37 ± 2.94	3.25 ± 1.81
Sixth group	0	3.96 ± 1.65	8.61 ± 3.5	2.24 ± 1.27

The secretion of gastric acid, which is a key factor regarding the healing of stomach ulcer, is reduced by using anti-acids, muscarinic and H2-receptor antagonists, and use of proton pump inhibitors [15]. In this study, ranitidine, which is an H2 receptor inhibitor and used widely as an acid inhibitor [15], was used as a standard control.

Murakami S. et al. demonstrated that tannic acid inhibits the hydrogen potassium ATPase in the stomach. Therefore, it can reduce gastric acid secretion and lead to positive effects on improving the treatment of gastric damage. In addition, the tannins, which are abundant in jaft extract are likely to reduce the gastric acid secretion with inhibitory effects on the hydrogen potassium ATPase [16]. These results are in accordance with our results in the present study, which reveals that pH in all the groups receiving the extract (first, second, and third groups) is higher than that in the control group (fifth group).

The histological or histomorphometric evaluation showed that the natural structure of the stomach has changed and pathological changes have been induced by stress. Increasing the interstitial fluid volume (edema), venous congestion, destruction of the mucosa and submucosal vessels would cause fine bleeding in the mucosal area. In addition, the apoptotic cells, reduction of the thickness of the gastric glands and mucosal folds of the stomach, infiltrations of neutrophils, and congestion of capillary on the mucosal surface are some evidence of inflammation induced by stress. The relationship between stress and stomach ulcer has been confirmed by several studies. Gastrointestinal injuries are caused by stress via several mechanisms, including inhibition of prostaglandin synthesis, production of oxygen free radicals, induction of apoptosis, reduction of gastric juice pH, increased volume of gastric juice, and increased secretion of neutrophils and nitric oxide production [17-19].

According to pathogen research, stomach ulcers are accompanied by a generation of reactive oxygen species (ROS) which is an essential factor involved in lipid peroxidation [20, 21]. The results show that a high amount of extract (752 mg/kg) significantly decreased the MDA production as a result of the increased antioxidant content of the extract.

## Conclusions

Plant extracts are a rich source of the most important natural antioxidants that protect the body against oxidative stress. Phenolic compounds with antioxidants properties play an important role in maintaining food production and protection to human health. Jaft extract contains abundant polyphenolic compounds and tannins and has several biological properties such as pharmacological properties, antioxidant activity, and inhibition of lipid oxidation [22-26].

## Conflict of Interest

The authors confirm that there are no conflicts of interest.
